# Multidisciplinary Collaboration in the Treatment of Patients With Type 2 Diabetes in Primary Care: Analysis Using Process Mining

**DOI:** 10.2196/jmir.8884

**Published:** 2018-04-10

**Authors:** Tania Conca, Cecilia Saint-Pierre, Valeria Herskovic, Marcos Sepúlveda, Daniel Capurro, Florencia Prieto, Carlos Fernandez-Llatas

**Affiliations:** ^1^ Computer Science Department School of Engineering Pontificia Universidad Católica de Chile Santiago Chile; ^2^ Department of Internal Medicine School of Medicine Pontificia Universidad Católica de Chile Santiago Chile; ^3^ Department of Family Medicine School of Medicine Pontificia Universidad Católica de Chile Santiago Chile; ^4^ Institute of Information and Communication Technologies Universitat Politècnica de València Valencia Spain

**Keywords:** process assessment (health care), interprofessional relations, primary health care, type 2 diabetes mellitus, data mining

## Abstract

**Background:**

Public health in several countries is characterized by a shortage of professionals and a lack of economic resources. Monitoring and redesigning processes can foster the success of health care institutions, enabling them to provide a quality service while simultaneously reducing costs. Process mining, a discipline that extracts knowledge from information system data to analyze operational processes, affords an opportunity to understand health care processes.

**Objective:**

Health care processes are highly flexible and multidisciplinary, and health care professionals are able to coordinate in a variety of different ways to treat a diagnosis. The aim of this work was to understand whether the ways in which professionals coordinate their work affect the clinical outcome of patients.

**Methods:**

This paper proposes a method based on the use of process mining to identify patterns of collaboration between physician, nurse, and dietitian in the treatment of patients with type 2 diabetes mellitus and to compare these patterns with the clinical evolution of the patients within the context of primary care. Clustering is used as part of the preprocessing of data to manage the variability, and then process mining is used to identify patterns that may arise.

**Results:**

The method is applied in three primary health care centers in Santiago, Chile. A total of seven collaboration patterns were identified, which differed primarily in terms of the number of disciplines present, the participation intensity of each discipline, and the referrals between disciplines. The pattern in which the three disciplines participated in the most equitable and comprehensive manner had a lower proportion of highly decompensated patients compared with those patterns in which the three disciplines participated in an unbalanced manner.

**Conclusions:**

By discovering which collaboration patterns lead to improved outcomes, health care centers can promote the most successful patterns among their professionals so as to improve the treatment of patients. Process mining techniques are useful for discovering those collaborations patterns in flexible and unstructured health care processes.

## Introduction

### Background

Type 2 diabetes mellitus (T2DM) is a chronic disease that can cause complications and serious health-related consequences [1]. This disease affects around 9.1% of the global adult population and is expected to reach 10% by 2040 [2]. In Latin America, the prevalence of T2DM is 9.4%, whereas in Chile it is 11.4% [2-5]. Consequently, T2DM constitutes a significant public health problem at the global level, affecting medium- and low-income countries to a greater extent [6]. As a consequence, considerable resources are allocated to its treatment, particularly in Chile [7].

When T2DM is not controlled, a series of complications can arise that impact the quality of life of the patient and which can increase their risk of mortality, for example, cardiovascular complications, diabetic retinopathy, peripheral neuropathy, and kidney failure [8,9]. It is possible to prevent these complications with good metabolic control [10] through the use of medication and an appropriate diet [1,11]. In the Chilean primary health care system, only 36% of the population with T2DM is classified as well-controlled or stable [4].

### Multidisciplinary Collaboration in Type 2 Diabetes Mellitus Care

Patients diagnosed with T2DM have multiple biomedical, psychological, and social needs that must be met in a coordinated manner by professionals from several disciplines. In this sense, multidisciplinary collaboration, which refers to joint work conducted by professionals from different disciplines who interact around a particular patient, has become critical for enhancing clinical outcomes [4,6,12-14]. Although clinical protocols establish treatment guidelines, health care processes for each patient vary and often deviate from standard indications [15]. The organization and composition of a treatment team can be an influential factor in patient evolution, as well as in the coordination between the different disciplines. Evidence suggests that the most successful interventions in relation to chronic diseases include certain key functions being carried out by nonphysicians, and interventions by well-integrated teams have been linked to greater patient satisfaction [16]. In the particular case of Chile, there is a significant shortage of physicians in primary health care [17]. Understanding how to organize multidisciplinary collaboration can facilitate the design of more efficient and effective treatment protocols.

Information systems record data related to services provided to patients. Process mining is a relatively new research discipline that has been used in health care to extract knowledge from information systems to analyze process design [18,19]. The algorithms developed in this discipline create graphical representations of models from the real execution of processes, which can be easily understood by individuals from a wide range of disciplines [20]. These models frequently demonstrate that reality differs to the perceptions, opinions, and beliefs held by parties directly involved in health care processes [21]. Process mining also facilitates the analysis of processes from an organizational perspective, which may help improve the understanding of how collaboration occurs within treatment teams [22].

### Research Goals

This study seeks to verify whether it is possible to determine certain patterns of collaboration using data from electronic clinical records (ECR) and to study if these patterns are related to the clinical outcomes of patients. Accordingly, we propose a methodology based on the application of process mining tools to analyze collaboration between health care professionals (HCPs) to (1) Identify collaboration patterns in the treatment of patients with T2DM in primary care, that is, the distinct interaction networks within the treatment teams and (2) evaluate the performance of the discovered patterns, confirming whether they relate to the clinical evolution of patients (represented by glycated hemoglobin, HbA_1c_ measurements). This approach uses information that is already being recorded in the relevant health care institutions and therefore, does not require the collection of new data.

### Related Work

#### Process Mining Applied to Health Care Processes

The inherent variability of health care processes has been addressed in a number of different ways. Some traditional control-flow discovery algorithms help to understand the different pathways that can be executed on a model and to distinguish the most common behaviors by managing the thresholds that indicate the frequency of activity sequences. The heuristic miner and fuzzy miner algorithms have been used to identify and study the main flow of the model based on data from the information systems of a hospital in Seoul, South Korea [23]. However, this approach generates a single model and, to discover different behaviors, it is necessary to test distinct thresholds, which can result in the analysis of unstructured processes becoming particularly complex. Another approach for creating simpler models for unstructured processes involves grouping several low-level activities with the same name at a higher level [24]. The proposed procedure is useful when the event log consists of large amounts of different activities and the traces differ not only in the sequence of activities but also in terms of the presence or absence thereof.

A different perspective is to create groups of patients according to certain preselected characteristics and to subsequently generate models for each group to capture the variability of the associated health care processes [25]. Once these groups of patients have been established, it is possible to generate models to represent the clinical flow followed by patients, including their progression across departments, specialists, and types of medical appointments, or over the natural course of an illness. This method has been applied to patients with T2DM using variables associated with related complications, including those concerning HbA_1c_, blood pressure, and cholesterol [26]. The results were used to analyze the circulation of the different groups and the probabilities of passing from one state to another.

Similar to the previous approach, other studies have successfully generated several simple models to represent highly flexible processes by applying different clustering techniques before the execution of discovery algorithms. The purpose of this additional step is to ensure that the logs with highly variable records become more manageable by grouping cases according to behavior similarity. Sequential clustering has been used during log preprocessing to identify regular behaviors, process variants, and exceptional cases [27]. While sequential clustering groups traces according to sequences of similar activities [28], trace clustering provides a set of grouping techniques based on distance that seeks to differentiate traces according to certain characteristics such as the frequency of activity occurrence, the number of events, or the number of events executed by each resource in a trace [29].

#### Collaboration in Medical Teams

Several studies have addressed the issue of collaboration in the treatment of patients with T2DM. One qualitative study from the patient perspective found that patients with T2DM and asthma consider that, to obtain the best outcome, it is necessary to receive treatment from a multidisciplinary team, despite them stating that they did not require such a team for their own treatment [30]. Another study found patients were satisfied with collaborative treatment [31]. No relationship between collaboration and clinical outcome was found in either of these studies.

Conversely, quantitative studies have given rise to evaluations of collaboration by considering the evolution of HbA_1c_, hospitalization costs, and readmission rates as clinical outcomes. One study found that the hospitalization costs and readmission rates decreased as the health care team became more integrated in terms of collaboration between physicians [32]. T2DM patients receiving treatment from multidisciplinary teams achieved better outcomes than those that did not, demonstrating improvements in HbA_1c_, *low-density lipoprotein* cholesterol, and an increased use of statins, as well as progress in statin and antiplatelet therapy [33]. Furthermore, differences have been identified in outcomes related to HbA_1c_, blood pressure, and cholesterol according to a report compiled by treating professionals [34]. A controlled study into the 2-year treatment of geriatric patients with T2DM by a multidisciplinary team, in comparison with a control group that received no collaborative treatment, found differences in the outcomes during the second year of collaborative treatment [35].

The organization and composition of the treating team can be an influential factor in patient evolution, as well as in terms of the coordination between the different clinical disciplines present within the team. For example, in one study, referrals to T2DM educators and dietitians were minimal, even among overweight and obese patients [12].

#### Process Mining, Social Network Analysis, and Collaboration

In the health context, some studies have used social network analysis to deepen understanding of the organization of professionals in patient care. It should be noted that although the methodology presented in this paper differs from the perspectives outlined in the previous section, the latter should not be discounted. The techniques can complement one another.

One study used visual graphics to demonstrate the structure of the referral networks and appointments that link physicians, defining four physician subgroups with similar referral, appointment, discussion, and attention coverage patterns [36]. The role of each physician was classified as the emitter, transmitter, or receiver according to the proportion of interactions that he or she initiated in relation to those initiated by others. A similar analysis was undertaken to gauge the structure of a team of nurses [37]. A further approach was based on the analysis of egocentric networks [38] by studying the network formed around a central actor and the actors with whom he or she interacts [39]. In this instance, the relationships between the secondary actors were not specified. Networks were used to analyze referrals and counter referrals among nurses and other disciplines to understand how they collaborate with other professionals and their contribution to multidisciplinary care in a primary health care setting. Accordingly, one important factor considered in this paper is referral flow, that is, whether this flow is unidirectional or bidirectional.

### Study Setting and Context

To better understand the context of this study and frame the implications of our results, next, we briefly describe the primary health care system in Chile.

The Chilean health care system is composed by both public and private insurers. The law mandates that each employed person must pay at least 7% of their income to a health insurer. Approximately 74.4% of the population is insured by the public system, and people who earn less than minimum wage, as well as children, students, and unemployed individuals, have free health care in the public provider network [40].

Primary health care centers, called Centro de Salud Familiar (CESFAM), are the first point of contact of users with the public health care network. These centers use a family medicine model in which patients are grouped into zones that treat at most 10,000 patients with a multidisciplinary team of health care workers. CESFAM treat acute morbidities that may be solved or referred to a more complex center and chronic morbidities that require periodic assessment, for example, diabetes, hypertension, and chronic pulmonary disease. The Chilean Ministry of Health establishes a treatment protocol for each of these conditions, published as a clinical guideline (eg, [4] for T2DM). The main problem faced by the CESFAM is a lack of resources, namely, not having enough HCPs. In Chile, the average number of physicians per 1000 inhabitants is 1.9, whereas for member states of the Organization for Economic Co-operation and Development it is 3.3 [17].

The Chilean Ministry of Health established in 2005 a program that prioritizes a set of 80 health conditions, guaranteeing timely and free access to treatment [40]. T2DM is one of such conditions. This, along with the Ministry guidelines for T2DM treatment, means that for beneficiaries of the public health care system, treatment is homogeneous: all patients have access to the same protocols and medications.

This paper is based on historical information from patients diagnosed with T2DM, collected in three CESFAM. These three centers are located in low income, high social vulnerability districts of Santiago, Chile. Overall, they have treated an average of 8000 people per year over the last 5 years, and approximately 30% of patients treated on an annual basis have T2DM. All three centers use the same flowchart, based on the clinical guidelines [4], to determine treatment for T2DM patients. The data collected correspond to the period from 2012 to 2016.

## Methods

### Data Source

After obtaining institutional review board approval, the dataset was extracted from the information system used in the three health care centers. Its database stores information related to patients, including their appointments, diagnoses, and test results. For each visit to the health care center, the system records the date, type of appointment, and the professional in charge of the episode. Importantly, this work only considers patients with T2DM and activities associated with periodic cardiovascular appointments (Cardiovascular Periodic Appointment, CVPA) that are performed by specialists from the professional triad team consisting of physician, nurse, and dietitian. Every time one of these professionals completes a CVPA, they must specify the discipline and approximate date of the patient’s next appointment.

The percentage of HbA_1c_ was selected as a metric to represent patient evolution. The HbA_1c_ test measures the glycemic history of the patient over the preceding 120 days [41] and is one of the tests used to monitor diabetic patients. The frequency of the test depends on the state of compensation of the patient, the treatment used, and medical judgment. Although the specific treatment objectives should be individualized for each patient, the American Diabetes Association recommends that the goal of therapy should be to reduce HbA_1c_ below 7%. For values higher than this, the clinical guidelines of the Chilean Ministry of Health clinical guidelines and the internal guidelines of the health care centers included in this study establish two categories of decompensation for patients: moderately decompensated, for values between 7% and 9% (included) and highly decompensated, for values higher than 9% [4]. The date on which a patient undergoes a test and its result are both logged in the records.

### Patient Selection

A total of 3369 patients with T2DM were identified across the three health care centers. Subsequently, to measure their respective evolution, we included individuals who had at least two recorded HbA_1c_ test results. In total, 2843 patients met these conditions.

To isolate external factors that might influence a patient’s evolution beyond the clinical team’s collaboration patterns, we included diabetic patients with no comorbidities or diabetes-related complications and good adherence to prescribed appointments and tests. We used the diabetes complication severity index and the chronic illness with complexity index count as measures of comorbidities and complications [1,42,43] and an interval under 4 months between the prescribed appointment and the actual appointment as a reasonable proxy for adherence.

Adherence to follow-up appointments is important for the evolution of patients, as through them professionals can intervene in the habits and self-care of the patient [44]. Greater rates of missed appointments are associated with significantly higher HbA_1c_ measurements [45]. Moreover, if patients do not show adherence to their treatment, the effectiveness of treatment is compromised, and they might develop complications [46]. In general, the diabetic population presents a low adherence both to medications and timely attendance to scheduled appointments [47]. To isolate the influence of the adherence to appointments, those patients who do not adhere to the HbA_1c_ tests were excluded. Nevertheless, a margin of time must be considered to determine that the patient attended the appointment on time [48]. The protocol of the health care centers stipulates that patients with higher states of decompensation should undergo more regular HbA_1c_ tests. In the context of the period under analysis, the following was considered acceptable by the health care centers studied: that patients in a state of compensation took the HbA_1c_ test up to 1 year after their last measurement, that patients who were moderately decompensated did the same after up to 6 months, and that highly decompensated patients did so after up to 3 months.

Given the context of the health care centers studied, in particular, their scarce resource availability, their restrictions for taking appointments (in general, patients cannot schedule appointments more than 1 month in advance), and the availability of hours for taking exams, it is normal that there is a delay that goes beyond the responsibility of the patient. To address these restrictions that depend on the health care center, a tolerance of up to 4 months for taking the HbA_1c_ test was considered. This time frame was discussed with and suggested by the HCPs.

Finally, patients who were tested for HbA_1c_ at intervals greater than those established for the clinical protocols according to their degree of compensation, considering a 4-month tolerance, were not considered in the analysis. This restriction ensures more complete information and greater consistency in terms of data evolution because the longer the time elapsed between tests, the more difficult it becomes to determine the variability in terms of patient compensation during that period. Of the 579 patients with neither severe conditions nor comorbidities, 319 had acceptable levels of adherence for inclusion in this study.

**Figure 1:**
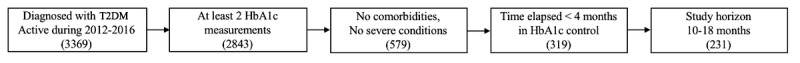
Criteria applied during patient selection process. T2DM: type 2 diabetes mellitus; HbA_1c_: glycated hemoglobin.

To normalize the period of study for all included cases, this paper considered a horizon of one and a half years to analyze the impact of multidisciplinary collaboration on the treatment of patients. The first measurement of HbA_1c_ that is available for a patient marks point zero of the period of study. To determine the end of the period, a subsequent HbA_1c_ measurement was sought as close as possible to 18 months after point zero. A tolerance period of 8 months was considered before and following the year-and-a-half mark, that is, the final measurement included had to fall within a range running from month 10 to month 26 (18±8), factoring in the possibility that other previous measurements may have been taken during this period. Of the 319 patients, 231 had a minimum acceptable study period of 10 months. As the study analyzed the response of the patient to the intervention and organization of the triad of professionals within a defined time frame, detailed information related to the complete evolution during the lifetime of the patient was not required. Figure 1 shows the patient selection process.

Regarding the sample, 133 out of 231 patients were men (57.6%), and 98 out of 231 were women. Overall, 50% (115/231) were aged 60 years or above, and 81% (187/231) were aged 50 years or above (average age 59.7; range: 20-89). The mean amount of CVPAs was 4.8 per patient (range: 1-15). Table 1 outlines this information.

**Figure 2:**
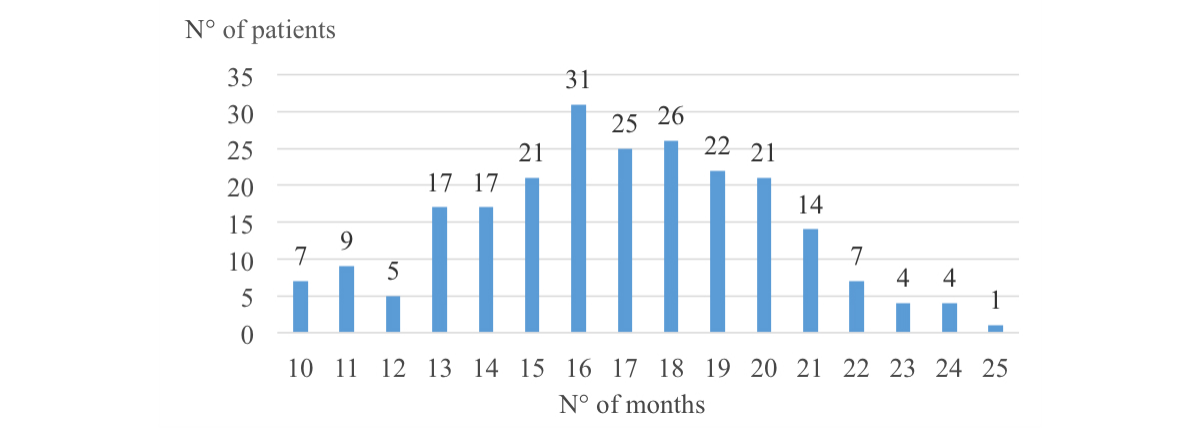
Periods of study used in the analysis.

Figure 2 outlines the duration of the periods of study considered. The X-axis shows the number of months included in the analysis, whereas the Y-axis shows the number of patients related to the corresponding horizon.

### Collaboration Through Network Analysis

Clinical records were used to build a log, with the following information for each CVPA: the ID of the patient, a time stamp, and the relevant attending discipline (physician, nurse, or dietitian). The time stamps were used to identify the sequence or order in which the disciplines intervened.

*Definition 1 (discipline log)*. *Let V represent the set of the three disciplines: physician (P), nurse (N), and dietitian (D), and H*={ *h*_1_,..., *h*_n_} *the set of patients. Let c*_hi_*be the sequence of disciplines in V who attend to patient h*_i_*. We define L, the discipline log, as L*={ *c*_hi_}∀ *h*_i_∈ *H.*

With information from the discipline log, a collaborative network can be created to show the relationship between the clinical disciplines in the treatment of patients. Specifically, a collaborative network related to a group of patients is defined as a directed graph in which the nodes refer to different clinical disciplines that intervene in the treatment of the disease, whereas the arcs represent the existing derivations among the disciplines in the case of each patient. It should be noted that following a CVPA of a patient, the professional may assign the subsequent appointment to either a professional from a distinct discipline or from the same discipline. Therefore, the graphs generated could include self-loops.

*Definition 2 (collaborative network). Let V represent the set of the three disciplines: physician (P), nurse (N), and dietitian (D), and H*={ *h*_1_,…, *h*_n_} *the set of patients. A collaborative network will be the graph G*^W^*=(V, E), where V={P, D, N} is the set of distinct disciplines that constitute part of the treatment of the patients in H and E ⊆* *{V × V} are the arcs that represent all the different derivations that occur when considering all the patients of set H.*

**Table 1:** Description of the studied population.

Variable	Average (SD)
Age	59.7 (12.6)
Years with type 2 diabetes mellitus	4.6 (3.8)
Number of glycated hemoglobin (HbA_1c_) measurements	3.7 (0.95)
Number of cardiovascular periodic appointments	4.8 (2.3)

**Figure 3:**
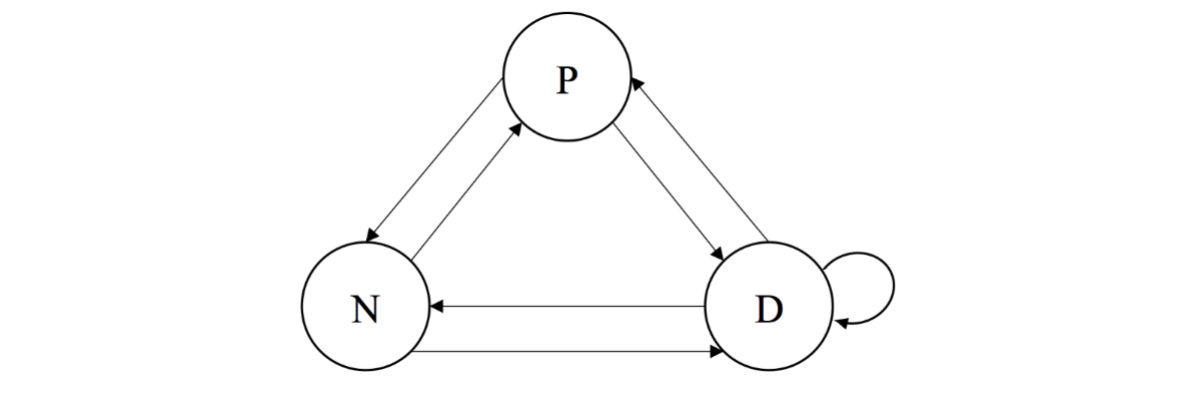
Collaborative network model. P: physician; N: nurse; D: dietitian.

Figure 3 shows an example of a collaborative network that represents the multidisciplinary structure of treatment received by a group of patients. In this case, the three disciplines make referrals among themselves, and the dietitian (D) makes self-referrals for certain CVPAs.

Three metrics were defined for both the nodes and the arcs of the collaborative network:

*Participation index:* the proportion of CVPAs performed by a specific discipline in relation to the total number of CVPAs. It is calculated for each discipline (node). It ranges from 0%, which represents no participation of the discipline in the treatment, to 100%, whereby all appointments were undertaken by the particular discipline. This value can be interpreted as the prominence of a clinical discipline with regard to the patient intervention.

*Self-referral index:* the proportion of CVPAs that are referrals to the same discipline in relation to the total number of CVPAs that a particular discipline refers in total. It is calculated for each discipline (self-loops). It ranges from 0%, which represents no self-referrals made by the discipline, to 100%, whereby all referrals made by the professionals of one discipline are to the same discipline.

*Referral index:* the proportion of CVPAs that one discipline refers to a different discipline in relation to the total number of CVPA referrals made by that particular discipline. It is calculated for each pair of disciplines (arcs between different nodes). It ranges from 0%, which represents no referrals to the other discipline, to 100%, whereby all referrals made by the professionals of one discipline are to the other discipline. This value can be interpreted as the level of support among different disciplines.

### Pattern Identification

The process mining algorithm selected for discovery was PALIA, implemented by the Institute of Information and Communication Technologies (ITACA) of the Universitat Politècnica de València, Valencia, Spain [49], which was applied using the PALIA Web [20] application. PALIA Web is a process discovery application created for the analysis of flexible and unstructured workflows. This tool was chosen because it receives an event log as input and outputs visualizations that are easy to understand for people who are not experts in process mining. In addition, it has filters that can be applied to the data before performing discovery, including, for example, trace clustering for the creation of different models based on groups of patients showing similar behavior.

The first step to identify the distinct forms of treating patients was to apply the PALIA process mining algorithm to the discipline log, complemented by trace clustering. This included the use of the flow disintegration functionality, which groups similar traces (sequences of disciplines that attend to each patient), and the application of the PALIA algorithm to create a visualization of the different groups or trace clusters.

The PALIA algorithm was executed with the following parameters for the flow disintegrations: similarity of 15% and outliers of 3%. The similarity percentage indicates that by conducting trace clustering, individuals from the same group are unable to differentiate by more than 15% according to the measurement of dissimilarity used by the algorithm, which is based on a heuristic topological editing distance [50]. Therefore, individuals from distinct groups differed by more than 15%. Conversely, the percentage of outliers indicates the minimum proportion of individuals that can be grouped under a single cluster. If the algorithm identifies a smaller group than the one established under that parameter, those patients are grouped together with the outliers. In this case, the 3% identified is equivalent to 7 patients. In addition, a heat map was applied to the diagrams (arcs and nodes) with a scale of red to green, where red indicates high frequency and green low frequency.

### Clinical Outcome

The test used to monitor and control the state of the patient was HbA_1c_. The clinical guidelines of the Chilean Ministry of Health, used by the health care centers, propose three categories for HbA_1c_ values: below 7% (called *compensated*, the clinical goal for patients), between 7% and 9%, and above 9%, each corresponding to a different course of action and time frame for follow-up [4]. As in our data we had several measurements for each patient, we propose a segmentation of patients considering their temporal trend [51]. We tried several different segmentations with the help of HCPs until we achieved a four-segment categorization that is described below. The HCPs stated that the most interesting category of patients for them were patients with high HbA_1c_ who, within the time frame, managed to reach the clinical goal (below 7%).

**Figure 4:**
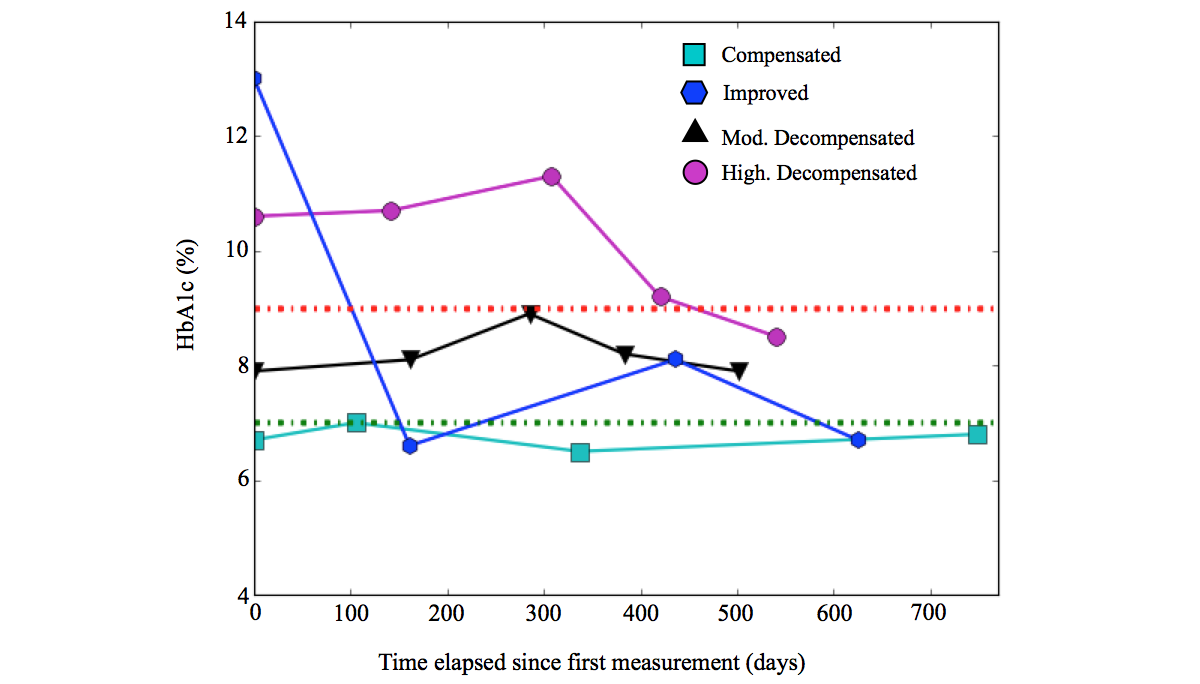
Example of the graphic representation of the clinical evolution of a patient of each segment. HbA_1c_: glycated hemoglobin.

Patients were separated into four segments according to the evolution of their HbA_1c_ results, as follows:

1. *Compensated*: patients with all measurements under 7%, or at the most one measurement between 7% and 9%, inclusive, but with an average in terms of all measurements under 7%, that is, it was accepted that these patients had exceeded the compensation limit once, but their average remained at a compensated level.
2. *Improved:* patients with a negative HbA_1c_ slope, and whereby their final measurement of the period was less than 7%, that is, regardless of their initial value; such patients showed a tendency to reduce their HbA_1c_ and end the study period in a compensated state.
3. *Moderately decompensated:* patients who did not reach or exceed 9% in any of their measurements, but who do not belong to the compensated or improved segments.
4. *Highly decompensated:* patients who recorded some measurements over 9%, and who do not belong to the improved segment.

For example, Figure 4 shows a graphical representation of one patient from each defined segment. The HbA_1c_ values of 7% and 9% are marked with dotted green and red lines, respectively. It can be seen that even though the compensated patient has one measurement equal to 7%, the average of his or her measurements is below 7%. In the case of the improved patient, despite that his or her HbA_1c_ increased at one point, the overall trend for HbA_1c_ was to decrease, and the patient completed the period of study in a compensated state. The moderately decompensated patient never exceeded 9% but failed to qualify as either improved or compensated. Finally, the highly decompensated patient spent the majority of the time with values in excess of 9% and failed to achieve compensated status by the end of the period.

### Statistical Analysis

The CVPA data of the 231 patients were collected, with a total of 1116 CVPAs. The analysis to study whether there is a statistically significant relationship between the identified patterns and patient evolution was undertaken using a proportion test. Fisher test was used when the evaluation sample proved to be too small. For each pattern, the proportion of patients who evolved in a specific manner was compared with those who evolved in the same manner in the total population studied. For all tests, the statistical significance was set to 0.05, and analysis was undertaken using R.

## Results

### Collaboration Patterns

**Figure 5:**
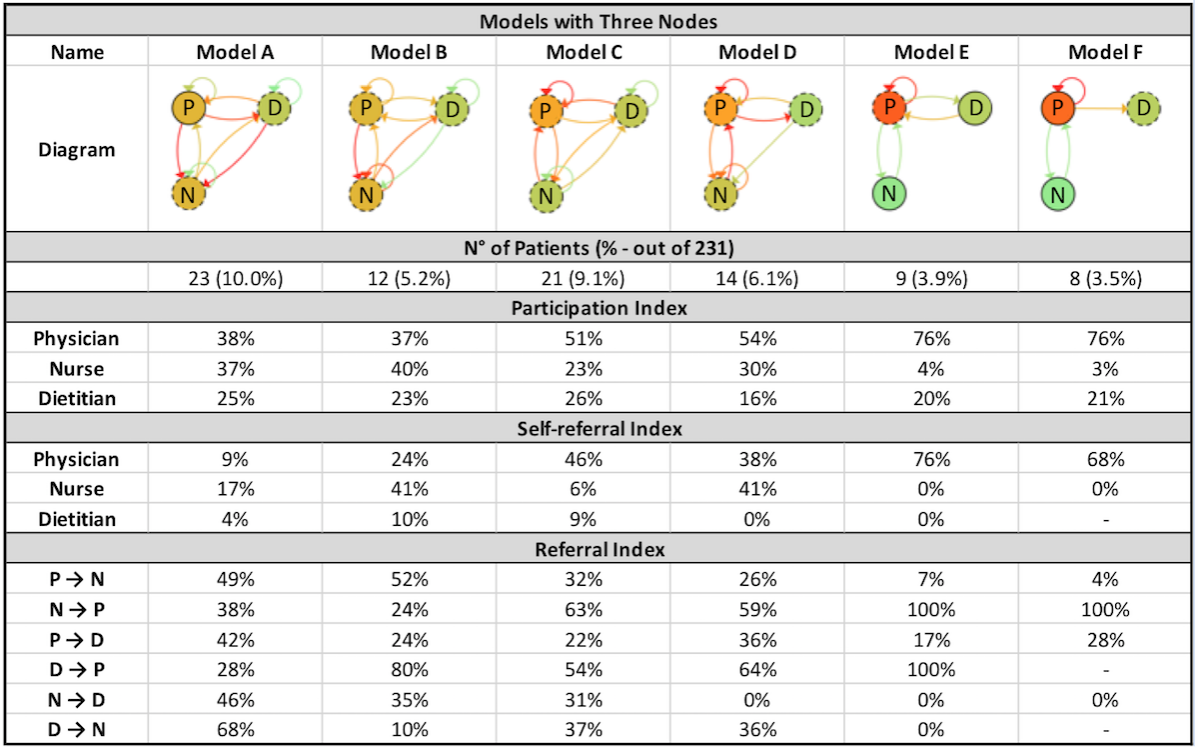
Models with three nodes created by the PALIA Web application with parameters: similarity=15% and outliers=3%. P: physician; N: nurse; D: dietitian.

**Figure 6:**
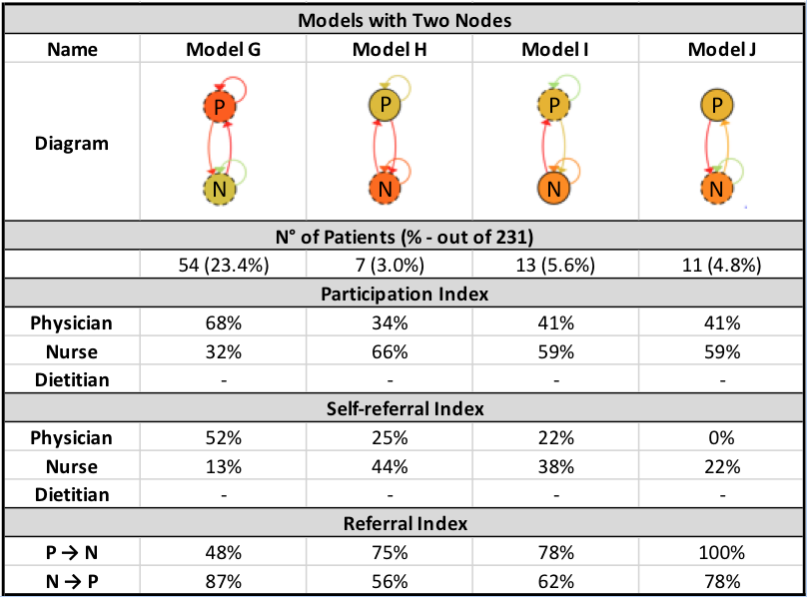
Models with two nodes created by the PALIA Web application with parameters: similarity=15% and outliers=3%. P: physician; N: nurse.

**Figure 7:**
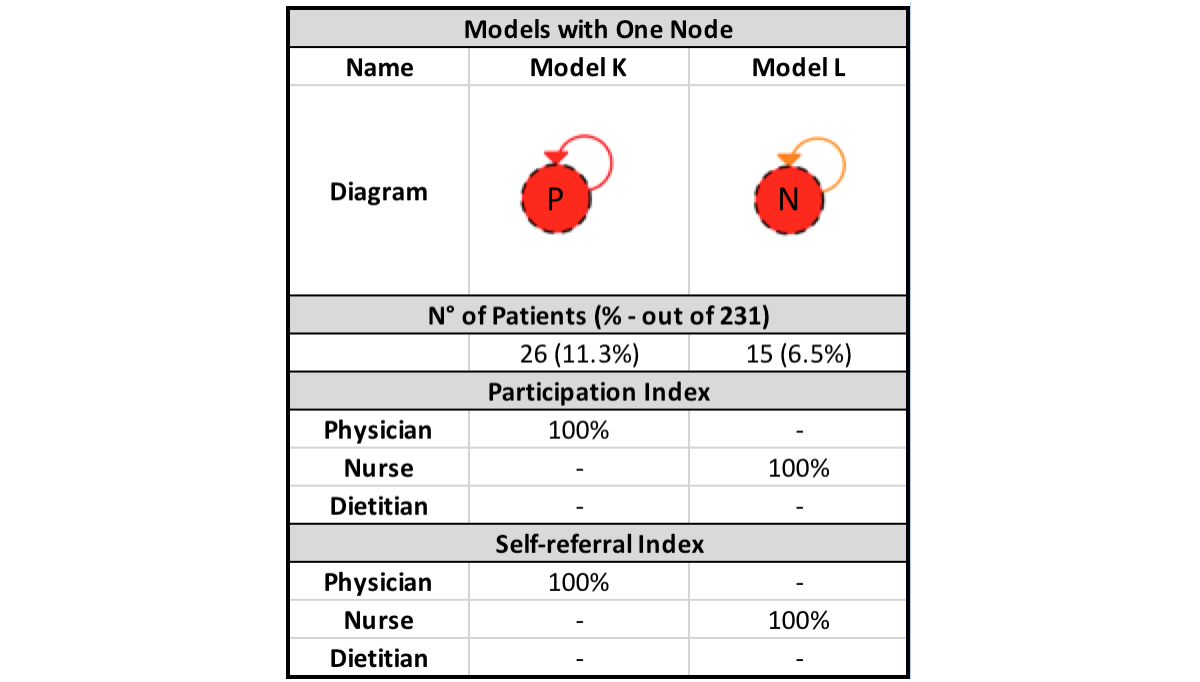
Models with one node created by the PALIA Web application with parameters: similarity=15% and outliers=3%. P: physician; N: nurse.

PALIA created 12 different models and 7.8% (18 patients out of 231) of outliers (see Figures 5-7). As is the norm in health care processes, there is a high variability in the results obtained. The most frequently occurring behavior is present in 23.4% of cases (54 out of 231), followed by 11.3% (26 out of 231) with respect to the second group, decreasing to 3.0% for the final group (7 out of 231). Of the 12 models, six (models A, B, C, D, E, and F) have three nodes, four (models G, H, I, and J) have two nodes, and two (models K and L) have only one node.

By reviewing the models in greater detail, certain similarities between the identified behaviors can be observed. To identify the collaboration patterns, differences and similarities regarding the participation and self-referral indexes were analyzed for the 12 clusters created by the algorithm. This analysis was conducted separately according to the number of nodes present (disciplines that participated in the intervention) in each model.

There are two groups of patients that are treated by only one discipline during the entire period of study. One of these is treated solely by a physician (model K) and another solely by a nurse (model L). Both behaviors were classified under one pattern we called *self-contained*, as, in this instance, only a single discipline attends to the patient.

Conversely, in the models with two nodes, it is possible to observe that clusters G and H have one node that takes the lead in treatment, with a participation percentage that exceeds 65% in both cases (68% for the physician and 66% for the nurse, respectively). In addition, the leader makes self-referrals in approximately half of all their CVPAs (52% and 44%, respectively) and refers the other half to a distinct discipline. In turn, the second node plays an important ancillary role in relation to the first by referring the majority of their CVPAs to the discipline leader (over 70% of cases) while making very few self-referrals. These behaviors are classified under the *tacit leader* pattern, as one discipline has a greater participation because the other discipline refers the majority of their cases to the former. In particular, it can be observed that the physician is the leader in model G, whereas the role of leader is performed by the nurse in model H.

In the other two clusters with two nodes (clusters I and J), evident similarities also enable to group them into a single pattern. The participation index is the same in each node for both clusters, and the participation index for both disciplines are in the range 50%±10% (59% and 41%), that is, the disciplines participate in an equitable way. By reviewing the referrals of the CVPAs, it can be seen that the level of self-referral is lower than in the aforementioned cases. Upon receiving a CVPA, each discipline prefers to refer the patient to the other discipline (over 60% of cases in each model). This pattern was called *shared*, as the participation of both disciplines is more equitable, with no clear leader, and whereby referrals among different disciplines are more prevalent than self-referrals.

Subsequently, the diagrams with three nodes underwent comparison. In clusters A and B, even though the dietitian participates to a lesser extent, the three disciplines have a more equitable participation according to their participation indexes. The node with the highest participation has a participation index of 40%. Therefore, there is no single discipline that acts as leader. There is interaction across all disciplines regardless of the direction of the interaction. In general, both clusters work in an integrated way and make referrals in a more equitable manner than the rest. Consequently, these clusters are grouped under a single pattern called *participatory*.

Clusters C and D are characterized by the physician occupying the central role in the collaboration, with a participation index of 51% and 54%, respectively, compared with the nurse and dietitian who have a lower participation index. However, it can be seen that there is more integration in cluster C than in cluster D, in which there is almost no interaction between nurse and dietitian (in either direction). Rather, the nurse makes self-referrals in the majority of the CVPAs and refers almost no cases to the dietitian, compared with cluster C. Therefore, they are deemed two distinct patterns. Cluster C is called *equitably centered*, as the physician is at the center of collaboration and the other two disciplines participate equitably. The physician occupies the role of the sole leader, as he or she self-refers a significant proportion of cases (46%, compared with 6% for the nurse and 9% for the dietitian). The cluster D is identified as a *hierarchically centered* pattern, as the nurse occupies the role of secondary leader after the physician by self-referring a significant portion of CVPAs. Furthermore, there is almost no interaction between nurse and dietitian.

Finally, clusters E and F are related in that the physician in both possesses almost complete control over all treatment. The physician presents a participation index that exceeds all other cases (76% in both clusters), which can be explained by the high self-referral index the discipline has (over 65%). In both clusters, the main interaction is between physician and dietitian, whereas the nurse’s participation index is below 5% in both clusters. Clusters E and F have been grouped under a *self‑referred leader* pattern. The seven identified patterns are summarized in Table 2.

### Clinical Outcome

By applying the aforementioned segmentation by clinical outcome to the 231 studied patients, four segments of patients were obtained. These segments are outlined in Table 3.

**Figure 8:**
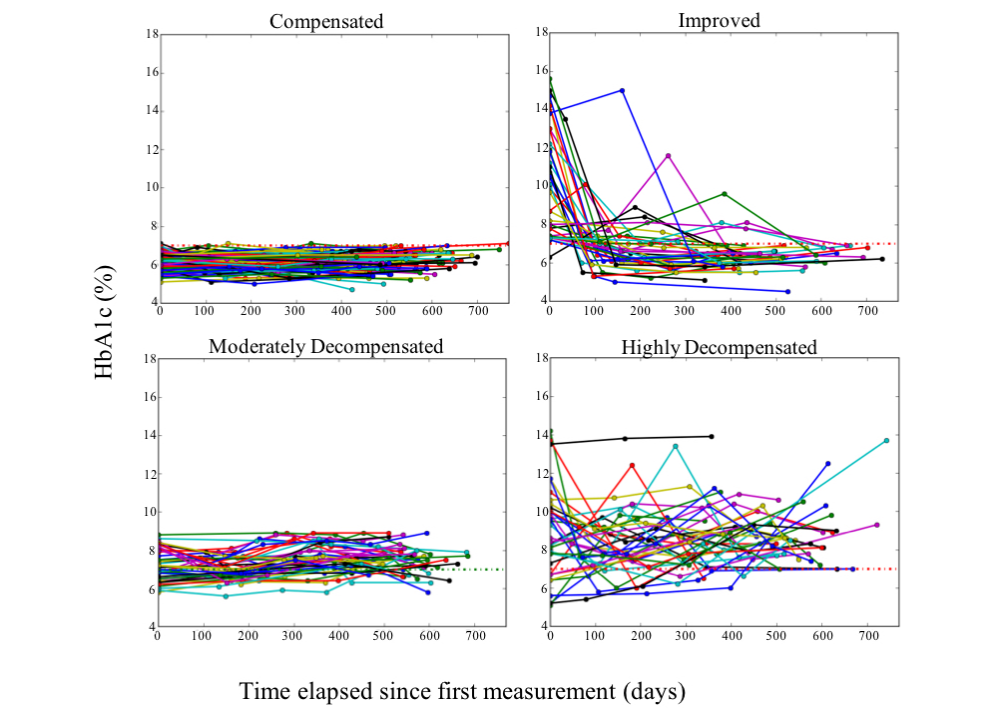
Clinical evolution of the patients in the different segments. HbA_1c_: glycated hemoglobin.

Figure 8 shows the evolution of the segments, displaying the values of the HbA_1c_ test for each patient over time. Each patient is represented by a line that corresponds to the value of his or her respective tests. The X-axis shows the time elapsed since the first measurement of each patient according to the period of study (which is not the same calendar date for all patients).

### Relationship Between Collaboration and Clinical Outcomes

The final step of the proposed methodology is to conduct a statistical analysis to evaluate whether there is a statistically significant relationship between the different patterns identified and the evolution of patients. A proportion test was performed, and Fisher test was used when the evaluation sample proved to be too small. For each pattern, the proportion of patients who evolved in a specific manner was compared with those who evolved in the same manner in the total population studied. For all tests, the statistical significance was set to 0.05, and analysis was undertaken using R.

In performing the proportion test, patients grouped under each collaboration pattern were considered as different subpopulations. For each subpopulation, the proportion of patients who evolved in a specific way in accordance with the four aforementioned compensation segments was calculated. As the number of patients is different in each subpopulation, the previous proportions were compared with the proportions of the total population. Table 4 outlines the overall frequencies and percentages obtained for each subpopulation and the total population, respectively.

The proportion test showed statistically significant differences for certain patterns vs the total population studied. The greatest difference was observed on patients treated under the self-contained pattern, in which 24% more remained compensated compared with the total population (73% vs 49%, *P*<.01). The second difference occurs in the participatory pattern, in which a lower proportion of highly decompensated patients is recorded compared with the total population (3% vs 16%, *P*=.03). Finally, treatment under the self‑referred leader pattern shows the worst result, with 19% more patients highly decompensated than the total population (35% vs 16%, *P*=.05).

### Evaluation of Professionals

The results obtained were initially presented to a primary care physician. The physician noted that the self-contained and participatory patterns coincided with her experience. The relationship of compensated patients with the self-contained pattern may be understood as the following: if a patient remains in a stable condition, it is more common for fewer disciplines to provide the medical attention. Conversely, the participatory pattern provides evidence that suggests the importance of having a multidisciplinary team that oversees patient care and also supports the significant role played by the dietitian in the treatment process, as they are the main promoters of change in terms of patient lifestyles.

After this preliminary evaluation, we evaluated the results with three different groups of primary care physicians (one for each of the health care centers involved in the study). During each session, one of the researchers presented the results and answered questions. Then, the physicians filled out an informed consent form and answered a questionnaire aimed at understanding whether our results matched their experience and gathering their perceptions, observations, and concerns about the results. Finally, we conducted a brief discussion in which participants were free to voice their opinion, and one researcher took notes.

**Table 2:** Identified collaboration patterns.

Pattern	Description
Self-contained	Only one discipline (either nurse or physician) intervenes in patient treatment.
Tacit leader	Two disciplines, nurse and physician, one of whom is the leader of the treatment.
Shared	Two disciplines, without a leader. Each discipline refers the majority of their cardiovascular periodic appointments to another discipline.
Participatory	Three disciplines participate equitably. There is no leader.
Equitably centered	Three disciplines, in which the physician is the leader. The nurse and the dietitian respond primarily to the physician, but they also interact among themselves (to a lesser extent).
Hierarchically centered	Three disciplines, in which the physician is the leader. The nurse and the dietitian respond primarily to the physician, and they do not interact among themselves.
Self‑referred leader	Three disciplines, in which the physician has almost complete control over treatment, receiving only minimal support from the other disciplines, primarily the dietitian.

**Table 3:** Patient segments according to their glycated hemoglobin (HbA_1c_) evolution.

Segments	Patients, n (%)
Compensated	114 (49.4)
Improved	37 (16.0)
Moderately decompensated	45 (18.6)
Highly decompensated	37 (16.0)
Total	231 (100)

**Table 4:** Number of patients according to collaboration pattern and clinical evolution segment.

Segment	Compensated n (%)	Improved n (%)	Moderately decompensated n (%)	Highly decompensated n (%)	Total n (%)
Self-contained	30 (73)	3 (7)	4 (10)	4 (10)	41 (100)
Tacit leader	23 (38)	12 (20)	15 (25)	11 (18)	61 (100)
Shared	14 (58)	2 (8)	4 (17)	4 (17)	24 (100)
Participatory	17 (49)	10 (29)	7 (20)	1 (3)	35 (100)
Equitably centered	7 (33)	3 (14)	5 (24)	6 (29)	21 (100)
Hierarchically centered	5 (36)	1 (7)	3 (21)	5 (36)	14 (100)
Self‑referred leader	5 (29)	2 (12)	4 (24)	6 (35)	17 (100)
Outliers	13 (72)	4 (22)	1 (6)	0 (0)	18 (100)
Total	114 (49.4)	37 (16.0)	43 (18.6)	37(16.0)	231(100)

**Table 5:** Evaluation results (N=23).

Pattern and segment		Observed by, n (%)
**Pattern**		
	Self-contained	5 (22)
	Tacit leader	6 (26)
	Shared	11 (48)
	Participatory	20 (87)
	Equitably centered	14 (61)
	Hierarchically centered	10 (43)
	Self-referred leader	8 (35)
**Segment**		
	Compensated	20 (87)
	Moderately decompensated	21 (91)
	Highly decompensated	18 (78)
	Improved	21 (91)

**Table 6:** Statement results (N=23). T2DM: type 2 diabetes mellitus.

Statement	Agreement
The patterns describe the main ways of collaboration in T2DM^a^ treatment in this Center	4.4
The patterns allow a correct classification of the ways of collaboration in T2DM treatment in this Center	4.2
Knowing these patterns may allow a better treatment of T2DM in this Center	4.3
The segments describe the main behaviors of T2DM patients in this Center	4.2
The segments allow a correct classification of the groups of patients treated for T2DM in this Center	4.3
It would be useful to treat differently patients classified in each segment	4.4

In total, 23 physicians participated in the evaluation. First, the participants stated whether they had observed each pattern and each segment. They were also able to name patterns and segments that had not been identified by the research. Then, participants answered a series of statements in a 5-point Likert Scale and several open questions (eg, “How useful are the relationships between patterns and segments?”). Some answers are provided below, translated from Spanish. A summary of the results is presented in Tables 5 and 6.

The participants stated that, in their experience, treatment of T2DM patients was participatory or equitably centered. One participant wrote, *I was surprised by the existence of a self-contained pattern.* They did not identify additional patterns, but did state the existence of other interventions in their centers, for example, educational workshops (to teach patients about how to handle T2DM), as well as other factors that may explain that a patient is always treated by a physician (One patient wrote as follows: *The disobedient pattern. Patients that go back to a physician although they were explicitly told to go to another professional [or that reject referrals]*).

Regarding the patient segments, these were more commonly identified by the participants—all of the segments had been observed by at least 18 out of 23 (78%) of participating physicians. When asked whether other segments were missing from our proposal (according to their experience), 4 mentioned the *worsened* segment, and 4 mentioned a *fluctuating* segment (which are both covered by the highly decompensated segment).

Overall, the evaluation by HCPs was positive. The physicians mentioned that the results could help them improve their protocols, patient treatment, and the management of human resources. Some mentioned that as there was no causal link established, they could not be certain that these changes would bring improvement.

## Discussion

### Principal Findings

The application of process mining techniques to the ECRs of the health care centers enables the analysis of the collaboration among HCPs. The advantage of the chosen algorithm is that it creates models that are easy to understand for HCPs. With these visualizations, the professionals in question may be able to view the work undertaken in the health care centers and comprehend how their protocols are actually taking place.

Leveraging the availability of data to control and improve processes can facilitate finding deviations from established protocols. One concrete example of this is the noncompliance with the norm that establishes appointments across three disciplines over the course of a year. It can be seen that 36.8% (85 out of 231) of patients had appointments with two disciplines during the period of study, whereas 17.7% (41 out of 231) with only one. Understanding how professionals collaborate may be useful in allocating resources according to existing requirements. It should be noted that to monitor processes, it is necessary to establish metrics that are aligned to that which professionals are seeking to control and improve.

In particular, the use of clustering techniques by graph topology facilitated overall management of the variability inherent to health care processes to further understand the process. PALIA helped enable the observation of the variability with which patient care was undertaken and, within this variability, those characteristics that differentiate certain behaviors from others.

### Comparison With Prior Work

By contrasting clinical evolution with the identified patterns, a number of differences can be observed. Certain comparisons showed statistically significant differences, which may signal a relationship between the collaboration pattern and overall patient evolution. Specifically, the self-contained pattern has a higher proportion of compensated patients compared with the total population. It may be possible to explain this because compensated patients are treated by a sole specialist, and this type of medical appointment is generally sufficient for them to remain stable, given the state of their condition. Other papers have published results that correlate collaboration with clinical outcomes, as randomized collaboration studies vs a control group [33,35,52], or via classifications of collaboration [34]. However, these studies have been unable to provide objective evidence of the collaboration patterns by means of quantitative analysis and their correlation with patient evolution.

Treatment under the participatory pattern is positively associated with patients who experience improvements in their evolution, and it is also associated with a lower proportion of patients who remain highly decompensated. If the level of significance is relaxed to 0.1, both characteristics are statistically significant. This outcome contrasts with the findings of Uddin [32], whereby he considers only integration between physicians in his analysis, in contrast to this paper, which incorporates evaluation by discipline.

The hierarchically centered and self‑referred leader patterns are associated with more significant proportions of patients who are highly decompensated. This differs from the findings of Bosch [34], who has found no statistically significant differences between the types of hierarchical collaboration. The typology introduced by Bosch is based on qualitative analysis and is self-reported, which could produce a bias that explains the discrepancy. Teams working under these patterns may view themselves as providing treatments that are failing to reduce the number of decompensated patients to the desired extent. In the case of the self‑referred leader, this discrepancy is statistically significant. Conversely, the shared and equitably centered patterns show no statistically significant differences compared with the total population. Therefore, the conclusion is that these particular types of treatment approaches cannot explain the evolution of patients with T2DM. Finally, the tacit leader pattern shows a lower proportion of compensated patients when compared with the total population studied (*P*=.06). It can be inferred that this type of treatment focuses on patients who show some type of decompensation.

In light of the foregoing, it can be observed that the main difference between the patterns with positive and negative performance is a distribution of participation between the distinct disciplines, which in the best-case scenario relates to more equitable participation. It also relates to the more integrated interactions between different disciplines. More participatory forms of work function better than those in which there is just one leader with significant control over treatment. The correlation between treatments that are primarily controlled by the physician (self‑referred leader) and an increase in HbA_1c_, compared with self-contained treatment and its respective correlation with compensated patients, is particularly interesting. It could be reasoned that one specialist is sufficient to monitor the state of patients who are well controlled, whereas a multidisciplinary intervention may be more beneficial for patients whose conditions are more serious.

This paper presents preliminary empirical results in relation to the organization of health care teams and the response of their patients to T2DM treatment. In contrast to the findings of Bosch [34], whereby the way in which teams organized themselves was self-reported, in this paper the approach used involved obtaining data from ECRs to ensure the type of collaboration was more objectively verifiable. However, despite their use of a completely different methodology, certain similarities arose with the Bosch typification, which divided groups into group culture, developmental culture, hierarchical culture, rational culture, cultural balance, and team climate. The relationships identified between the patterns and the evolution of patients does not necessarily constitute a causal link. A relationship in the opposite direction may, in fact, be possible. Indeed, certain external factors and individual characteristics of the patients could affect comparisons. For example, lifestyles, compliance with clinical indications, amount of exercise, or nutritional habits are all factors that may impact the evolution of patients with T2DM.

### Limitations

The main limitation of the proposed methodology is that the analysis procedure and information used will be determined by the availability and quality of the data collected by information systems. The reception of incomplete and inconsistent data was one of the main problems faced. Although the professional expert in the field is the individual who is able to guide analysis objectives, the availability of data is the factor that determines the steps that must be taken to address these objectives. Some relevant data that may affect patient outcomes were not available, for example, some health care centers undertake education efforts in the form of workshops. Furthermore, there was no available information about medication adherence. Therefore, there may be certain steps described in this paper that require adaptation if they are to be extended to other cases with additional (or fewer) data. One available variable that was not used in the analysis was age (eg, the clinical goal for HbA_1c_ at the centers for patients over 80 is 8% instead of 7%).

A further limitation is that multidisciplinarity was only measured at the level of the cardiovascular team and did not include other types of professionals. Moreover, data were only analyzed at the discipline level regardless of the particular professional that provided care.

This study is based on data from 2012 onwards, so some results may not be representative to the present day. The clinical appointments included in this study relate to scheduled visits explicitly registered as CVPAs in the information systems. A comparison of collaboration between the different health care centers could also have been beneficial, although the proportion of patients in the related sample would have been extremely small to generate statistically significant results. In the future, it would be useful to execute this analysis using a larger sample. Finally, future studies are recommended to include the severity of the patient condition and their comorbidities as variables to measure patient evolution via changes in these indexes over time, rather than using them as filters.

### Conclusions

The use of process mining represents an opportunity for health care centers to understand how their processes are being executed and which forms of collaboration lead to improved outcomes. The definition of simple clinical-based segmentations is critical for facilitating the interpretation of results. The process mining tool used in this research, PALIA Web, allows to analyze how different HCPs collaborate in flexible and unstructured processes such as the treatment of T2DM patients. By combining trace clustering (to manage the diversity of patients present in the log) and collaboration pattern discovery techniques, it is able to identify several collaboration patterns among HCPs in the treatment of patients. It also allows to easily visualize the obtained collaboration patterns so that HCPs can interpret the differences between them. The methodology used in this study made it possible to analyze the relationship between these collaboration patterns and the clinical evolution of patients, so as to identify the most successful patterns. Finally, health care centers can then promote the most successful patterns among their professionals so as to improve the treatment of T2DM patients.

## Abbreviations

CESFAM: Centro de Salud Familiar
CVPA: cardiovascular periodic appointment
T2DM: type 2 diabetes mellitus
ECR: electronic clinical record
HbA _1c_: glycated hemoglobin
HCP: health care professional
